# The challenges of cancer during pregnancy

**DOI:** 10.1111/aogs.14835

**Published:** 2024-04-04

**Authors:** Kenny A. Rodriguez‐Wallberg, Karin Pettersson

**Affiliations:** ^1^ Department of Reproductive Medicine Karolinska University Hospital Stockholm Sweden; ^2^ Department of Oncology‐Pathology Karolinska Institutet Stockholm Sweden; ^3^ Department of Obstetrics and Gynecology Karolinska Institutet CLINTEC and Karolinska University Hospital Stockholm Sweden

Although more attention has been given recently to knowledge gaps regarding pregnancy‐associated cancer, development of solid evidence‐based clinical guidelines and improvements in healthcare for this patient group are still to come. The current data appear insufficient to make firm conclusions. Studies from Australia,^1^ Denmark^2^ and Canada^3^ have previously reported an increasing annual crude incidence rate of pregnancy‐associated cancer, thus we need to be better prepared for the management of an increasing number of such patients in the future. However, divergence among studies is worth noting as previous data indicate that incidence for this rare condition may also differ among different regions within one country.^4^ In this special issue of *AOGS* dedicated to cancer during pregnancy, several population‐based studies are reported. Population‐based studies provide the richness of data encompassing the whole population, including its diversity, but these are only possible in countries that have initiated population‐based registries in previous years, such as Australia, Canada and some European countries including the Nordics, that also have enough follow‐up time allowing the identification of outcomes of interest over time in these populations.

Among the reported population‐based studies in this issue, Lundberg et al. identified the risk factors for pregnancy‐associated cancer in Sweden, including both cancer during pregnancy or occurring within the first year post delivery, and found that increasing maternal age, a current trend in Western societies, was the strongest risk factor for the increasing incidence of cancer‐associated pregnancy.^5^ Another study from Australia reported by Fotheringham et al.,^6^ was dedicated to gynecological cancers diagnosed in pregnant women, and the authors found that for that cancer type, there was no increase in incidence over time in their country, perhaps reflecting effective cervical cancer screening and implementation of vaccine programs against human papillomavirus in recent years. This means that prospective improvements could be seen in future studies in many countries.

However, at present, cancer during pregnancy is still challenging in many ways, and the disease has been described as a rare condition. The greatest challenge is the lack of expertise, as most obstetricians, gynecologists and oncologists do not see or manage enough cases neither during their training, nor thereafter as specialists. Thus, collaborative work to increase the global knowledge and improve healthcare is urgently needed.

In 2018, Sweden initiated such collaborative work and produced the first Nordic Healthcare Program on Cancer during pregnancy, a multidisciplinary effort of obstetricians, midwives, oncologists, oncology nurses, hematologists, gynecological oncology surgeons, pediatricians, radiologists, psychiatrists and patient representatives from the whole country, coordinated by the Swedish Regional Cancer Centers (RCC).^7^ The RCC act as the support entity aiming at improving cancer care quality, and increasing the efficiency of healthcare resources use with a focus on the patient care pathway. The RCC follow the intentions of the national cancer strategy and the EU's cancer plan, aiming to meet the challenges and demands in tomorrow's cancer care through outreach and collaborative work, and the resulting guidelines are publically available to healthcare providers and the public.^7^

In the Netherlands, an earlier initiative has been reported with the establishment of an Advisory Board on Cancer in pregnancy in December 2012 to support physicians’ decisions and management. A total of 13 medical disciplines are represented on the board, and during less than 10 years the advisory group has provided 213 recommendations to 42 hospitals.^8^ The experience of consultations with the board was evaluated and judged positive in 2020 using a survey. In 2021 an International advisory Board on Cancer in Pregnancy (ABCIP) was established by the International Network on Cancer, Infertility and Pregnancy (INCIP), which currently provides advice to over 20 countries.

The additional challenges of cancer during pregnancy are also evident from the collection of a limited number of cases in the literature. Therefore systematic reviews and meta‐analysis are also needed, which is well illustrated in the studies by Marasciulo et al.,^9^ and Zhang et al.,^10^ in this special issue. Importantly, as noted in the study reported by Metcalfe et al.,^11^ the systematic exclusion of pregnant and lactating patients from clinical trials has largely contributed to maintainance of evidence gaps on how best to treat these patients. The reason for excluding pregnant and lactating patients from studies appears to be the fear of research‐related risks, which is currently a significant barrier to finding the best treatment approach to protect these patients from further complications.
